# Pentastatin-1, a collagen IV derived 20-mer peptide, suppresses tumor growth in a small cell lung cancer xenograft model

**DOI:** 10.1186/1471-2407-10-29

**Published:** 2010-02-01

**Authors:** Jacob E Koskimaki, Emmanouil D Karagiannis, Benjamin C Tang, Hans Hammers, D Neil Watkins, Roberto Pili, Aleksander S Popel

**Affiliations:** 1 Department of Biomedical Engineering, School of Medicine, Johns Hopkins University, Baltimore, MD 21205, USA; 2 Department of Chemical and Biomolecular Engineering, Johns Hopkins University, Baltimore, MD 21218, USA; 3 The Sidney Kimmel Comprehensive Cancer Center at Johns Hopkins, School of Medicine, Baltimore, MD 21231, USA; 4 Monash Institute of Medical Research, Monash Medical Centre, Victoria, Australia; 5 Roswell Park Cancer Institute, Buffalo, New York 14263, USA

## Abstract

**Background:**

Angiogenesis is the formation of neovasculature from a pre-existing vascular network. Progression of solid tumors including lung cancer is angiogenesis-dependent. We previously introduced a bioinformatics-based methodology to identify endogenous anti-angiogenic peptide sequences, and validated these predictions *in vitro *in human umbilical vein endothelial cell (HUVEC) proliferation and migration assays.

**Methods:**

One family of peptides with high activity is derived from the α-fibrils of type IV collagen. Based on the results from the *in vitro *screening, we have evaluated the ability of a 20 amino acid peptide derived from the α5 fibril of type IV collagen, pentastatin-1, to suppress vessel growth in an angioreactor-based directed *in vivo *angiogenesis assay (DIVAA). In addition, pentastatin-1 suppressed tumor growth with intraperitoneal peptide administration in a small cell lung cancer (SCLC) xenograft model in nude mice using the NCI-H82 human cancer cell line.

**Results:**

Pentastatin-1 decreased the invasion of vessels into angioreactors *in vivo *in a dose dependent manner. The peptide also decreased the rate of tumor growth and microvascular density *in vivo *in a small cell lung cancer xenograft model.

**Conclusions:**

The peptide treatment significantly decreased the invasion of microvessels in angioreactors and the rate of tumor growth in the xenograft model, indicating potential treatment for angiogenesis-dependent disease, and for translational development as a therapeutic agent for lung cancer.

## Background

Judah Folkman pioneered the field of tumor angiogenesis by demonstrating that solid tumors are dependent on their blood supply to grow and metastasize, thus placing the field of tumor angiogenesis at the center of cancer biology and therapeutics [1]. Lung cancer is responsible for the highest percentage of cancer deaths (~28%) in both men and women worldwide [2]. The number of annual deaths is over 160,000 in the United States, and annually there are over 215,000 newly diagnosed cases [3]. Angiogenesis has been well documented to play a role in lung cancer development and progression [4-6]. Clinical evidence of angiogenesis in lung cancer is abundant, manifested as an increase in intratumoral microvascular density [7]. The over-expression of vascular endothelial growth factor (VEGF) has also been correlated with rapid small cell lung cancer (SCLC) growth [8]. Although SCLC makes up only 20% of lung cancer cases, its characteristics have been described as extremely virulent - correlated with rapid cell growth, high resistance to chemotherapy, and low median survival [9]. These factors correlate with a shorter relapse-free and overall survival, indicating the necessity for comprehensive treatment.

We recently developed a bioinformatics-based approach to predict over 100 novel endogenous anti-angiogenic peptides [10]. An important class of peptides determined by this method was derived from the αIV, αV, and αVI fibrils of type IV collagen, designated tetrastatins, pentastatins, and hexastatins, respectively. The bioinformatics predictions were validated *in vitro *in cell proliferation and migration assays on human umbilical vein endothelial cells (HUVECs) [11]. Most peptides showed a significant degree of anti-angiogenic activity; the 20-mer peptide pentastatin-1 derived from αV fibrils demonstrated high activity in both cell proliferation and migration experiments. Based on these results we applied pentastatin-1 to an angioreactor-based directed *in vivo *angiogenesis assay (DIVAA), and to an *in vivo *NCI-H82 SCLC xenograft model. We demonstrate high activity in each of these assays, in addition to directly inhibiting proliferation of NCI-H82 SCLC cells and 3T3 fibroblasts *in vitro*, indicating strong potential for pentastatin-1 as a therapeutic agent for lung cancer.

## Methods

### Peptide synthesis and handling

The peptide pentastatin-1 (LRRFSTMPFMFCNINNVCNF) was synthesized using a solid-phase synthesis technique by a commercial provider (Abgent, San Diego, CA). The endogenous human and mouse sequences are identical for this peptide. The manufacturer provided HPLC and mass spectrometry analysis to guarantee >95% purity. The peptides were stored at -80°C in lyophilized form. Since pentastatin-1 is hydrophobic, it was solubilized using 10% dimethyl sulfoxide (DMSO) and water without any demonstrated effect on cell viability.

### WST-1 cell viability experiments

*In vitro *viability assays were completed with pentastatin-1 on NCI-H82 small cell lung cancer and mouse 3T3 fibroblast cell lines. NCI-H82 human SCLC cells were obtained from the laboratory of Dr. D. Neil Watkins (JHMI, Oncology). The cells were propagated in RPMI 1640 cell medium (Invitrogen, Carlsbad, CA) supplemented with 10% v/v fetal bovine serum, 10 mM of HEPES, 2 mM of L-glutamine, 1% v/v of pen/strep, 1.5 g/l of sodium bicarbonate, and 1 mM of sodium pyruvate. The cells do not attach to the flask, but grow in small floating colonies and are passaged by centrifugation (1,000 RPM, 5 min) without trypsinization and subsequently resuspended in fresh media. 3T3 mouse fibroblast cells were acquired from ATCC (Manassas, VA) and grown under standard conditions in Dulbecco's Modified Eagle Medium (DMEM) (ATCC) with 10% FBS and 1% pen/strep.

The effects of the peptide on the NCI-H82 and 3T3 cell viability were measured using the colorimetric cell proliferation reagent WST-1 (Roche, Indianapolis, IN). Approximately 2 × 10^3 ^cells were seeded per well in a 96-well microplate, centrifuged at 1,000 RPM for 5 minutes, and exposed for 3 days to peptide concentrations of: 3.2, 6.3, 12.5, 25, 50, and 100 μg/mL. Cells were tested in triplicate for each concentration. Microplates containing NCI-H82s were centrifuged at 1,000 RPM for 5 minutes to minimize loss of non-adherent cells before application of WST-1. As an experimental control, equivalent to normal cell viability, the cells were cultured without any agent in complete medium, containing growth factors and serum without any exposure to peptide.

### BrdU cell proliferation assay

Cell proliferation was measured by BrdU (5-bromo-2'-deoxyuridine) incorporation assay (Calbiochem/Merck, Whitehouse Station, NJ) on both NCI-H82 cells and 3T3 fibroblasts according to manufacturer's recommendations. Briefly, 10,000 cells/well were plated into 96 well plates in the presence of pentastatin-1 at 3.8, 15, and 60 μg/mL, and BrdU label (1:2000 dilution) for 24 hours. Plates were then washed, fixed with anti-BrdU antibody, and peroxidase goat anti-mouse IgG conjugate. Immunocomplex formation was measured using tetra-methylbenzidine solution, and the reaction terminated using 2.5 N sulfuric acid. The measured intensity is proportional to the amount of incorporated BrdU in the cells. Absorbance was measured at 450 nm using a Victor 3 V plate reader (PerkinElmer, Waltham, MA). Cells were tested at five wells per concentration.

### Directed in vivo angiogenesis assay (DIVAA)

DIVAA (Trevigen, Gaithersburg, MD) is a quantitative *in vivo *method of assaying angiogenesis. Silicone cylinders of 20 μl volume and 100 μm of inner diameter (angioreactors) were closed on one side, and filled with an extract of extracellular matrix containing VEGF and fibroblast growth factor (FGF) with or without premixed peptides as a control. These angioreactors were then implanted subcutaneously in the abdominal region of C57BL/6 mice, two angioreactors per animal, ventrally one per each side of the peritoneal cavity. Capillary sprouts originating from the host vessels invaded the extracellular matrix and formed vessels in the angioreactor. 12 days after the implantation, the mice were euthanized and angioreactors were removed. The extracellular matrix, containing the developed vasculature, was removed from the cylinder and endothelial cells were stained using FITC-Lectin, quantified by fluorescence at 510 nm emission with 485 nm excitation using a fluorescence plate reader (Victor 3 V, Perkin Elmer, Waltham, MA). The intensity of the signal is proportional to the number of endothelial cells contained in each of the angioreactors. The results were normalized to the mean of the experimental controls. Statistical significance was tested by the Student's t-test at *p *< 0.05 *.

### In vivo tumor xenograft model

Animal protocols were approved by the Animal Care and Use Committee at the Johns Hopkins Medical Institutions. Athymic nude mice were obtained from Harlan (Indianapolis, IN). The mice were 4-6 weeks of age and housed on a 12 hour light dark cycle with food and water provided ad lib. All mice were fed with a standard caloric diet for their age.

Mice were allowed to acclimate for one week prior to inoculation with NCI-H82 cells. NCI-H82 cells were grown under the same conditions for *in vitro *cell viability experiments. Prior to inoculation, the cells were prepared in a solution containing 1:1 mixture of PBS and Matrigel (BD Biosciences, San Jose, CA). 1 × 10^6 ^cells in 200 μl volume were injected subcutaneously in the right flank area of the mice. Tumors were inspected 5 to 6 days after inoculation. The tumor inoculation efficiency was approximately 75%.

Following growth incubation of 6-7 days, tumor size volume was estimated by measurement of tumor dimensions with digital calipers, and peptides were administered once per day for 12 days, intraperitoneally (i.p), at 5 mg/kg and 10 mg/kg. In most cases the initial tumor volume ranged from 150 mm^3 ^to 200 mm^3^. Equivalent volumes of 10% DMSO in water were injected as an experimental control. Separate controls of scrambled peptide sequences were also made to test that the anti-angiogenic efficacy of pentastatin-1 was sequence-dependent. The injections were continued for 12 days, with a total of 8 animals per group used for the experiments per peptide per concentration. The location and side of the injection were alternated every day. The higher dose of 10 mg/kg was repeated twice with similar tumor suppression and without a statistically significant change.

The tumor dimensions were measured every three days. We measured two dimensions for each tumor, a long and short axis. In some cases the tumors developed as two lobes, in which we considered the whole tumor as a single lobe with two associated axes. In order to estimate the volume we considered the tumors as prolate spheroids with volume equal to *(4/3)πab*^*2*^, where *a *is the measurement for the long semi-axis and *b *is the measurement for the short semi-axis.

### Statistical analysis

The average tumor size per condition and the standard error of the mean are reported over time. The statistical significance measured using the Student's *t*-test with significance defined at *p *< 0.05 * and *p *< 0.01 **. Statistical significance was tested among the peptide condition and the experimental control in addition to the scrambled peptide.

### Immunohistochemistry

Immediately following the sacrifice of the mice, tumors were excised and stored in a zinc-based fixative (BD Biosciences, San Jose, CA) for 10-14 days, and sent to the JHMI Immunohistochemistry Core Facility for paraffin wax-embedding and processing. After deparaffinization and rehydration, 5 μm cross sections were treated overnight at room temperature with a monoclonal rat anti-mouse CD31 (PECAM-1) IgG antibody (1:100) (BD Pharmingen, San Jose, CA), which specifically recognizes an epitope on the surface of endothelial cells. Secondary antibody incubation was made using biotinylated rabbit anti-rat IgG antibody (1:200) for one hour, and lightly counterstained with hematoxylin.

Similar analysis was made for the apoptotic marker cleaved (activated) caspase-3. After deparaffinization and rehydration of slides, primary antibodies were applied at dilutions of 1:400 for cleaved caspase-3 (rabbit polyclonal antibody; Cell Signaling Tech., Boston, MA, Cat. #9661), diluted in antibody dilution buffer (ChemMat, San Dimas, CA, Cat.#ADB250) and incubated at 14 hours at 4°C. Primary antibodies were detected using the Power Vision Plus HRP-polymer detection system (Leica Microsystems, Bannockburn, IL, Cat.#PV6119) per manufacturer's instructions. DAB chromogen (Sigma Chemicals, St. Louis, MO, Cat.#D4293) was applied to develop the secondary detection reagent. Slides were counterstained with hematoxylin and mounted on cover slips.

All histological samples were digitized, then processed using Aperio Image Scope Software (Aperio Technologies Inc., Vista, CA), and quantified using FRiDA software (Johns Hopkins University, Baltimore, MD). For the CD31 antibody staining 100% represents the mean of the experimental controls. Apoptotic cell counts were made per frame at 20× magnification using FriDA. Each peptide concentration was compared to the control and scrambled peptide equivalent using the Student's *t*-test at *p *< 0.05 * and *p *< 0.01 **.

## Results

**Figure 1:**
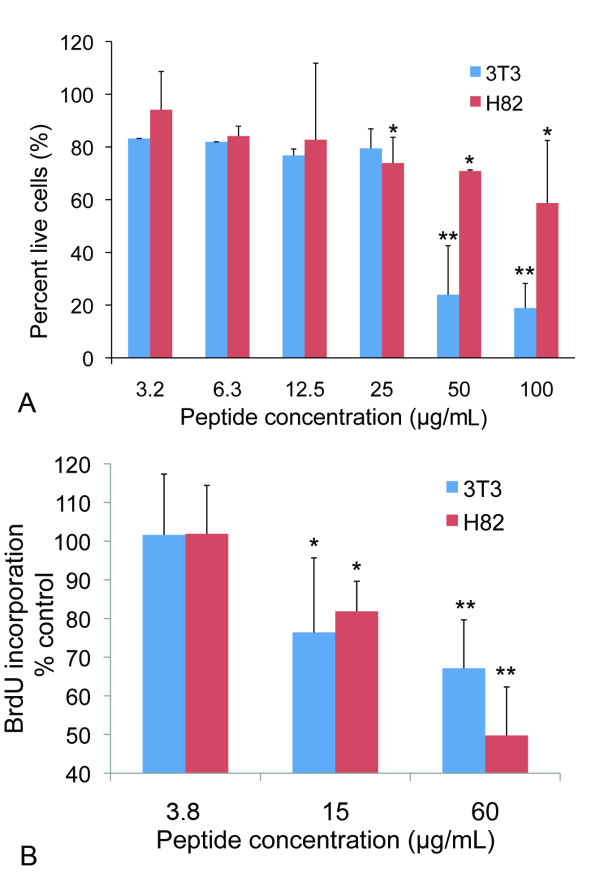
**In vitro cell viability assay**. **A**. NCI-H82 small cell lung cancer cells (H82) and 3T3 fibroblasts (3T3) were plated at 2,000 cells/well. Pentastatin-1 peptide was applied at increasing concentrations up to 100 μg/mL, and incubated for three days. Cell viability was determined using the WST-1 colorimetric agent, and scaled to the untreated experimental control. Differences between H82 and 3T3 treatments are statistically significant as determined by the Student's t-test at *p *< 0.05 * and *p *< 0.01 **. **B**. BrdU incorporation for NCI-H82 small cell lung cancer cells and 3T3 fibroblasts. Cells were plated at 10,000 cells/well with pentastatin-1 at concentrations of 3.8, 15 and 60 μg/mL in the presence of a BrdU label for 24 hours. BrdU incorporation was detected by the BrdU-antibody peroxidase conjugate as a measurement for cell proliferation by DNA synthesis. Pentastatin-1 decreases cell proliferation by 50% for H82 and 33% for 3T3 at 60 μg/mL. Statistical significance is determined at *p *< 0.05 * and *p *< 0.01 ** by Student's *t*-test.

*In vitro *cell viability experiments show pentastatin-1 decreases NCI-H82 SCLC and 3T3 fibroblast viability at increasing concentrations (Fig. 1A). Pentastatin-1 decreases NCI-H82 cell viability by 26% at 25 μg/mL, and to a maximum decrease of 42% at 100 μg/mL in comparison to the untreated controls. The effect on 3T3 fibroblasts is significant with a decrease of 20% at 25 μg/mL, and a maximum effect of an 81% decrease on cell viability at 100 μg/mL measured by the WST-1 colorimetric reagent. There is a statistically significant difference from the control beginning at concentrations of 25, 50, and 100 μg/mL for NCI-H82 SCLC, and 50 and 100 μg/mL for 3T3 fibroblasts. Similarly, we show that pentastatin-1 inhibits cell proliferation through DNA synthesis by incorporation of BrdU label (Fig. 1B). Upon incubation of peptide for one day, pentastatin-1 inhibits proliferation by 50% for NCI-H82 and 33% for 3T3 fibroblasts at 60 μg/mL.

**Figure 2:**
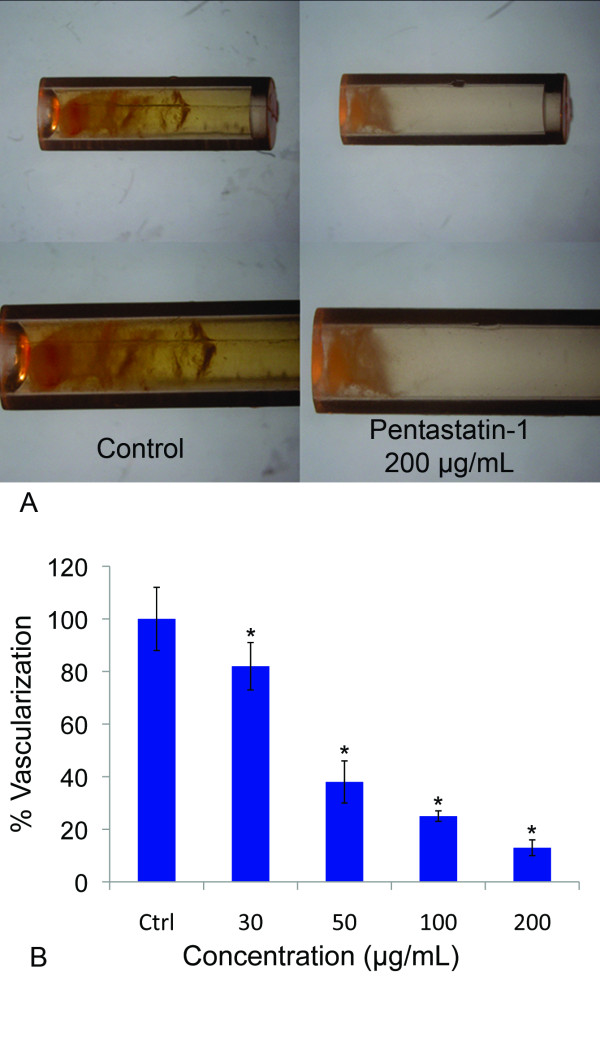
**angioreactor-based *in vivo *assay**. **A**. Directed *in vivo *angiogenesis assay (DIVAA) showing silicone tubes with and without application of pentastatin-1 at 200 μg/mL. Angioreactors were implanted subcutaneously into the abdominal region of C57BL/6 mice for 12 days, and vessels allowed to infiltrate. **B**. Peptides were mixed at concentrations of 30, 50, 100, and 200 μg/mL with Matrigel (BD Biosciences, San Jose, CA), and vascularization assayed and scaled where 100% represents the mean of the untreated controls (Ctrl). Results are significantly less than controls at *p *< 0.05 *.

We show the application of pentastatin-1 in two *in vivo *angiogenesis assays. Fig. 2 shows the results of the DIVAA. The invasion of vessels is markedly reduced in the angioreactors containing the peptide (Fig. 2A), shown for the control and the highest peptide concentration of 200 μg/mL. The percent vascularization of the tubes decreases with increasing peptide concentration, shown in Fig. 2B. At 30 μg/mL the vascularization decreases by 19%, and this trend continues to a 67% decrease for 50 μg/mL, and an 88% decrease at 200 μg/mL in comparison to the experimental control. Results are statistically lower from the control at *p *< 0.05 beginning at concentrations of 30 μg/mL.

**Figure 3:**
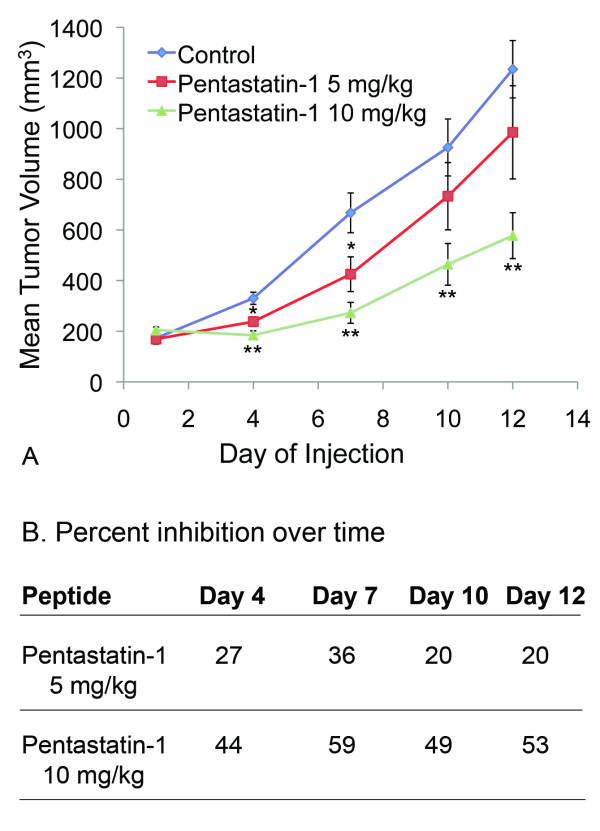
**NCI-H82 small cell lung cancer xenograft in nude mice**. **A**. Mean tumor volume reported over time for the NCI-H82 SCLC xenograft model. Peptides were administered intraperitoneally, once per day, at two separate concentrations of 5 mg/kg or 10 mg/kg in comparison to a PBS-treated control and a scrambled peptide equivalent (not shown). Pentastatin-1 significantly suppressed growth for all days at 10 mg/kg by Student's t-test at p < 0.01 ** and demonstrated a dose response in vivo. **B**. Percent inhibition from day 1 reported over time for pentastatin-1 at 5 mg/kg and 10 mg/kg.

In the tumor xenograft experiment we follow the mean tumor volume of pentastatin-1 treated xenografts (Fig. 3A), along with the experimental control and scrambled peptide over time for two doses of 5 mg/kg and 10 mg/kg. The control tumors grow rapidly to a final mean volume of 1,235 mm^3 ^on day 12. The lower dose of 5 mg/kg statistically suppresses tumor growth by 20% by day 12 (Fig. 3B). Beginning on day 4 the average tumor volume of the 10 mg/kg is statistically lower than the experimental control, and this significance was maintained until the termination of the experiment for *p *< 0.01. The higher dose of 10 mg/kg increases the suppression of the tumor more than the 5 mg/kg concentration to a 53% inhibition by day 12. The scrambled peptide is used to verify the correlation between amino acid content and tumor suppression is sequence-dependent, and is not statistically different from the control tumors at any point in the experiment (data not shown). The percent inhibition for each condition over the 12 days for the experiment is summarized in Fig. 3B. Experiments were terminated on day 12 as the controls had reached maximum values outlined in the ethical care and use protocol.

**Figure 4:**
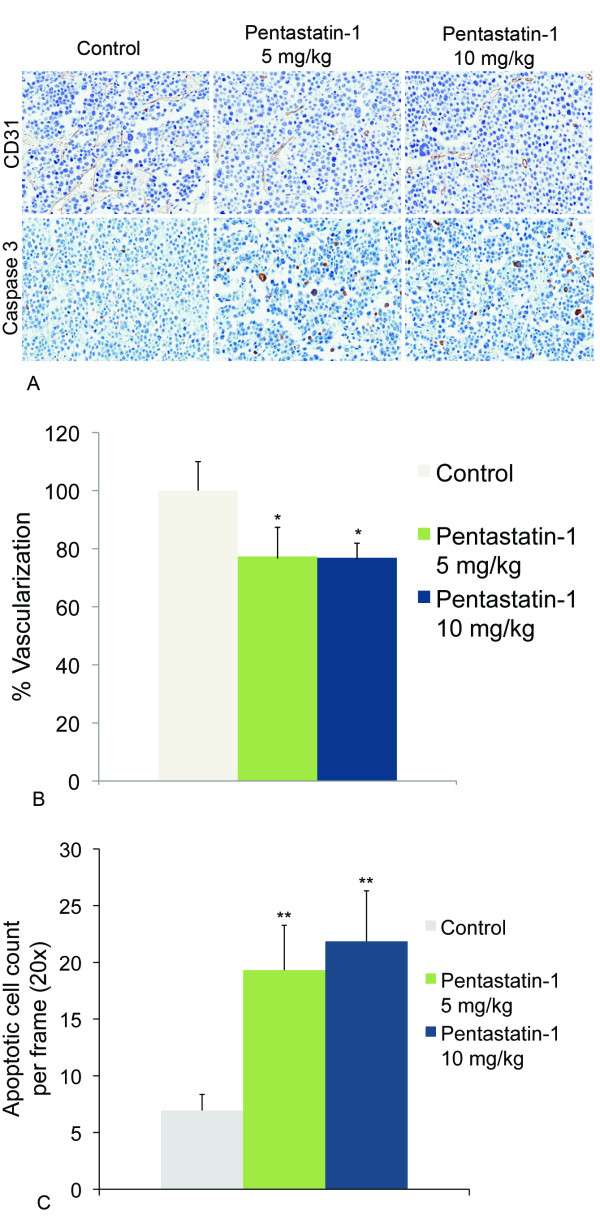
**CD31 antibody staining for microvascular density**. **A**. Immunohistochemistry showing CD31 as a marker for endothelial cells, and caspase-3 antibody staining for apoptotic cells in the control, and pentastatin-1 at 5 mg/kg and 10 mg/kg as a marker for endothelial cells and blood vessel density. **B**. Quantification of microvascular density using CD31 antibody. Blood vessels are stained in tumor sections using the CD31 antibody, and endothelial cells quantified by pixel intensity representing the quantity of endothelial cells as a percentage of the control. Three sample tumors for each condition with eight cross sections were quantified. Pentastatin-1 at 5 mg/kg and 10 mg/kg showed a statistically significant 23% and 24% decrease in microvascular density, respectively, from the control at *p *< 0.05 *. **C**. Quantification of the mean number of apoptotic cells per frame at 20× magnification for the PBS-treated control, and pentastatin-1 at 5 mg/kg 10 mg/kg. Controls show an average 7 cells/frame, while pentastatin-1 at 5 mg/kg shows 19 cells/frame and pentastatin-1 at 10 mg/kg show an average of 22 cells/frame. Results are statistically significant at *p *< 0.01**.

Fig. 4A shows the effect of pentastatin-1 on the microvascular density of the tumors, visually, as CD31 is a marker for endothelial cells. Similar caspase-3 immunohistochemistry reveals the presence of apoptotic cells within the tumor microenvironment. The experimental control contains more vessels than the peptide-treated tumors, and this is verified quantitatively in Fig. 4B. Both concentrations at 5 mg/kg and 10 mg/kg are statistically different from the experimental control, showing a 23% decrease for 5 mg/kg and 24% decrease for 10 mg/kg in vasculature compared to the control. The scrambled peptide is not statistically different from the control, but is different from the peptide-treated tumors. Fig. 4C shows the number of apoptotic cells per frame at 20× for the PBS-treated controls, and pentastatin-1 at 5 mg/kg and 10 mg/kg. Controls contained a mean number of 7 apoptotic cells/frame, while pentastatin-1 contained 19 and 22 apoptotic cells/frame at 5 mg/kg and 10 mg/kg, respectively.

## Discussion

Currently, there is a high unmet need for treatment of small cell lung cancer as upwards of 95% of patients diagnosed with the disease will die within five years [9]. A number of pharmaceutical agents are in clinical trials for SCLC [12], e.g. tyrosine kinase inhibitors imatinib (Gleevec, Novartis, East Hanover, NJ), and gefitinib (Iressa, AstraZeneca, Wilmington, DE), along with the anti-VEGF antibody agent bevacizumab (Avastin, Genentech/Roche, San Francisco, CA) [13]. Although peptide-based therapies have development obstacles to overcome such as short *in vivo *half-lives and degradation by proteases, they are also advantageous due to low toxicities, high selectivity, and potential to enhance existing cytotoxic therapies [14].

Collagen IV forms the bulk of the structure of the vascular basement membrane, and is known to contain several cryptic fragments with anti-angiogenic activity [15]. Several collagen-derived peptides are being tested in pre-clinical models such as tumstatin [16] and celingitide in glioblastoma [17]. We have shown that pentastatin-1, a collagen IV derived 20-mer peptide, is effective in a preclinical model of SCLC. The high potency of pentastatin-1 in the disease-independent DIVAA assay shows a strong potential for application to various angiogenesis-dependent disease models, including cancer. The suppression of tumor growth in the SCLC xenograft demonstrates specific application to lung cancer, and potential for clinical development.

We have previously shown pentastatin-1 decreases HUVEC migration and viability *in vitro*. Similarly, we show pentastatin-1 effectively inhibits viability of NCI-H82-SCLC cells and 3T3 fibroblasts by WST-1 colorimetric agent, and inhibits synthesis of DNA by BrdU incorporation indicating the peptide targets multiple cell types in addition to being anti-angiogenic. Previously published xenograft work shows pentastatin-1 has low toxicity measured by H&E staining of vital organs in severe combined immunodeficient (SCID) mice [18]. We have also shown that pentastatin-1 targets β_1 _and β_3 _integrins, which are expressed on endothelial cells [10], and are also expressed on several SCLC cell lines [19] including NCI-H82s [20], indicating likely targets for pentastatin-1 to bind on this cell line.

In our xenograft model we show pentastatin-1 has a monotonic dose response and suppresses tumor growth at both 5 mg/kg and 10 mg/kg, while decreasing the microvascular density and increasing apoptotic cell count in response to the peptide application. At the higher dose the peptide suppresses tumor growth significantly with an inhibition of 53%. Although the tumors grow and reach a size larger than the initial size when peptide was administered, the peptide still effectively suppresses the tumor growth when used as monotherapy. The scrambled peptide equivalent shows that the peptide's activity is sequence-dependent, as it is not statistically different from the control. The presence of β_1 _and β_3 _integrin receptors on endothelial cells is consistent with the decrease in microvascular density, as the peptide targets these receptors, while a lower microvascular density is correlated with a decrease in tumor development and growth.

## Conclusions

Type IV collagen contains several cryptic fragments with anti-angiogenic properties. We previously identified several anti-angiogenic fragments through a bioinformatics-based methodology, including pentastatin-1, which showed high potency to *in vitro *HUVEC proliferation and migration assays. Small cell lung cancer is very resistant to chemotherapy, and has a high mortality rate indicating the need for comprehensive treatment. The disease has been shown to be angiogenesis-dependent. Based on previous *in vitro *experiments, we selected pentastatin-1 for application to the angioreactor-based DIVAA, and a small cell lung cancer xenograft model using nude mice. The peptide has shown to be effective in a pre-clinical model for limiting tumor growth, and has the potential for translational research in lung cancer.

## Competing interests

The authors declare that they have no competing interests.

## Authors' contributions

JK and MK conducted and analyzed all *in vitro *and *in vivo *experimental data. JK prepared the manuscript for submission. BT, HH and RP supervised xenograft inoculation and provided experimental and technical assistance regarding data analysis. DNW provided NCI-H82 small cell lung cancer cells, and experimental guidance. AP conceived design of the study, supervised *in vitro *and *in vivo *assays, and provided useful discussion for data interpretation. All authors read and approved the final manuscript.

## Pre-publication history

The pre-publication history for this paper can be accessed here:

http://www.biomedcentral.com/1471-2407/10/29/prepub
